# Effects of Oleic Acid Addition Methods on the Metabolic Flux Distribution of Ganoderic Acids R, S and T’s Biosynthesis

**DOI:** 10.3390/jof8060615

**Published:** 2022-06-09

**Authors:** Meng-Qiu Yan, Xiao-Wei Su, Yan-Fang Liu, Chuan-Hong Tang, Qing-Jiu Tang, Shuai Zhou, Yi Tan, Li-Ping Liu, Jing-Song Zhang, Jie Feng

**Affiliations:** Institute of Edible Fungi, Shanghai Academy of Agricultural Sciences, National Engineering Research Center of Edible Fungi, Key Laboratory of Edible Fungi Resources and Utilization (South), Ministry of Agriculture, Shanghai 201403, China; yanmengqiu@sina.com (M.-Q.Y.); susu1822615@163.com (X.-W.S.); liuyanfang@saas.sh.cn (Y.-F.L.); tangchuanhong123@163.com (C.-H.T.); tangqingjiu@saas.sh.cn (Q.-J.T.); simonzsz@163.com (S.Z.); judy_1989317@126.com (Y.T.); l1228llp@163.com (L.-P.L.)

**Keywords:** *Ganoderma lucidum*, submerged fermentation-static culture, oleic acid, metabolic flux analysis, ganoderic acid

## Abstract

The effects of oleic acid addition methods on the metabolic flux distribution of ganoderic acids R, S and T’s biosynthesis from *Ganoderma lucidum* were investigated. The results showed that adding filter-sterilized oleic acid in the process of submerged fermentation and static culture is of benefit to the synthesis of ganoderic acids R, S and T. The metabolic fluxes were increased by 97.48%, 78.42% and 43.39%, respectively. The content of ganoderic acids R, S and T were 3.11 times, 5.19 times and 1.44 times higher, respectively, than they were in the control group, which was without additional oleic acid. Ganoderic acids R, S and T’s synthesis pathways (GAP), tricarboxylic acid cycles (TCA), pentose phosphate pathways (PP) and glycolysis pathways (EMP) were all enhanced in the process. Therefore, additional oleic acid can strengthen the overall metabolic flux distribution of *G. lucidum* in a submerged fermentation-static culture and it can reduce the accumulation of the by-product mycosterol. This study has laid an important foundation for improving the production of triterpenes in the submerged fermentation of *G. lucidum*.

## 1. Introduction

*Ganoderma lucidum,* a traditional medicinal mushroom included in Chinese Pharmacopoeia, has a history of more than 6800 years. It contains a variety of active components, such as polysaccharides, triterpenes and nucleotides. Among them, triterpenes have a wide range of pharmacological activities, such as antioxidant, anti-aging, anti-inflammatory and anti-tumor [1]. Triterpenes are lanostane derivatives, and lanosterol is their precursor. Lanostane-type triterpenes can be divided into tetracyclic triterpenes and pentacyclic triterpenes, according to their chemical structure; according to carbon atoms, they can be divided into C24, C27 and C30. In addition, they can be divided into ganoderic acid, ganoderol, ganoderal and ganolactone, according to different functional groups and side chains, with ganoderic acid and ganoderol as the main ones [2]. It has been found that ganoderic acid R, ganoderic acid S [3] and ganoderic acid T [4] are the main triterpenes in *Ganoderma lucidum* mycelium, which exhibit anti-liver cancer, anti-cervical cancer and anti-prostate cancer effects [5]. Triterpenes can be obtained by artificial cultivation and liquid submerged fermentation; liquid submerged fermentation is considered to be an effective way to accumulate triterpenes from *Ganoderma lucidum* due to its advantages of short cultivation time and the ease of controlling the culture conditions [6].

In recent years, there have been many studies on the acquisition of triterpenes from *G. lucidum* by submerged fermentation. However, due to a lack of understanding of the biosynthesis and the metabolism of triterpenes from *G. lucidum*, the application of key technologies, such as the metabolic regulation in the fermentation of triterpenes from *G. lucidum,* is limited. The anabolism of triterpenes in *G. lucidum* is complex, and the subtle differences in the fermentation process may cause great changes in the metabolic flux of products. Therefore, fermentation regulation is an important way to obtain a high yield of triterpenes. There have been reports that the addition of exogenous substances in the fermentation system could interfere with triterpene synthesis, for example, methyl jasmonate, phenobarbital, aspirin, copper ions and calcium ions could induce triterpene synthesis [7,8]. In addition, due to the aerobic fermentation of *G. lucidum* mycelium, in order to improve the oxygen concentration in the culture medium during its liquid fermentation, we found that adding monounsaturated fatty acid oleic acid in the fermentation process of *G. lucidum* mycelium could greatly increase the content of triterpenes in the mycelium [9]. Through the addition method of oleic acid in the fermentation process of *G. lucidum,* the mycelium was systematically optimized. The study showed that two ways of adding oleic acid, high-temperature-sterilized oleic acid and filter-sterilized oleic acid, had a great influence on the synthesis of triterpenes from *G. lucidum* [10].

However, there are few and insufficient studies on fungal triterpene anabolism at present. The existing studies on triterpene anabolism have mainly focused on plants. For many years, terpenes biosynthesis has been considered to start from the mevalonate pathway (MVP) [11]. It has been confirmed that the triterpenes of *G. lucidum*, like other terpenoids, were synthesized by the mevalonic acid biosynthesis pathway [12,13]. The upstream process from acetyl-coenzyme A to mevalonic acid and then to lanosterol is basically clear, but the further pathways of forming various triterpenes are still unclear. That is to say, after the synthesis of lanosterol, the carbocyclic skeleton must undergo a complex modification, and little is known about the specific steps of this process. The unclear downstream pathway of triterpenes synthesis greatly limits the application of metabolic engineering or pathway engineering in the submerged fermentation regulation of triterpenes. However, metabolic flux analysis may provide a good solution.

Metabolic flux analysis quantitatively describes the flow distribution of each branch in the metabolic network, which is helpful in analyzing the bottleneck in the synthesis process of target products, and it has great significance for understanding the intracellular metabolic regulation mechanism [14]. When using metabolic flux analysis, the metabolic reactions without branches can be combined into one reaction in the process of constructing a metabolic network. This makes it possible to combine the unknown reactions from lanosterol to triterpenes of different structures into one reaction by using this principle.

On the basis of the above analysis and of previous studies, metabolic flux analysis was used to analyze the effects of oleic acid addition methods on triterpenes’ metabolic biosynthesis during the liquid fermentation of *G. lucidum* mycelium. It provides guidance for the following modification of *G. lucidum* strains and the optimization of the fermentation strategy to promote the triterpenes yield.

## 2. Materials and Methods

### 2.1. Strains

Strain: The strain used in this study was *Ganoderma lucidum* G0023, which was preserved by the Agricultural Culture Collection of China (the edible fungi branch). It was maintained on potato dextrose agar (PDA) slants. The culture was inoculated and incubated at 26 °C for 10 days, then stored at 4 °C for approximately two months.

### 2.2. Medium and Culture Conditions

The seed medium contained (g/L) the following: glucose 30, yeast extract 4, KH_2_PO_4_ 1.5, MgSO_4_·7H_2_O 1.5 and pH 5.5. For the seed culture, three loops of agar culture, in pea-size, from a slant culture were inoculated into a 250 mL flask, with 100 mL seed medium and were cultivated for nine days in a reciprocal shaker at 120 rpm and 26 °C. The seed culture (10%, v/v) was inoculated into the fermentation medium.

The fermentation-static medium contained (g/L) the following: glucose 30, yeast extract 3, KH_2_PO_4_ 2, MgSO_4_·7H_2_O 2 and pH 5.5. For liquid submerged fermentation, the seed culture was inoculated at 10% into a 250 mL flask, with 100 mL seed medium. Culture conditions of temperature and agitation rate were fixed at 26 °C and 120 rpm, respectively. According to the literature and preliminary experimental data, 1.21% high-temperature-sterilized oleic acid (121 °C, 30 min) was added to the flask at 32 h; 1.35% filter-sterilized oleic acid (filtered with 0.22 μm microfiltration membrane) was added at 7 h; after 5 days submerged fermentation, the broth was transferred to static culture at 26 °C, and growth period after static culture for 15 days. The culture without oleic acid was used as control. All experiments were performed in triplicate.

### 2.3. Analysis Methods

Determination of dry weight of mycelial samples: 100 mL of fermentation broth were centrifuged at 15,000× *g* for 15 min, reserved supernatant, and the pellet was washed with distilled water 3 times and dried at 60 °C, until a constant weight was achieved. We calculated the dry weight of mycelium per liter of fermentation broth after precise weighing, measured in g/L. Three parallel experiments were set in each group, and the average value was measured [15].

Determination of glucose: The supernatant prepared in the determination of the dry weight of the mycelium was diluted and then determined by DNS method to calculate the content of glucose [16].

Determination of ergosterol: The dried mycelium was extracted with absolute ethanol, at the ratio of solid to liquid of 1:10. Ultrasonic extraction for 1 h, centrifuged at 8000× *g* for 5 min, and the supernatant was taken for high-performance liquid chromatography (HPLC) analysis. Chromatographic conditions: Agilent ZORBAX SB-Aq (150 × 4.6 mm, 5 μm) column was selected. The mobile phase was acetonitrile-acetic acid (0.01%) to water, with a ratio of 95:5, the flow rate was 1.0 mL/min, the column temperature was 30 °C, the injection volume was 10 μL and the analysis wavelength was 282 nm [17].

Determination of ganoderic acids R, S and T: The dried mycelium was extracted with absolute ethanol at the ratio of solid to liquid of 1:20, and then centrifuged at 8000× *g* for 5 min, with ultrasound for 1 h, and the supernatant was taken for detection. Chromatographic conditions: Agilent ZORBAX Eclipse C18 (4.6 × 250 mm, 5 μm) column was selected. The mobile phase was acetonitrile-acetic acid (0.01%) to water, with a ratio of 55:45, the flow rate was 1.0 mL/min, the column temperature was 30 °C, the injection volume was 10 μL and the analysis wavelength was 240 nm [18].

### 2.4. Statistical Analysis

The experimental data were analyzed by MATLAB R2018a software, plotted by Origin 2021 software and analyzed by Microsoft Excel 2019. The data averaged from three individual experiments are expressed as the mean ± standard error (SE). Error bars present the standard deviations from the means of triplicates.

## 3. Results

### 3.1. Effects of Oleic Acid Addition Methods on Ganoderic Acids R, S and T’s Biosynthesis

**Figure 1:**
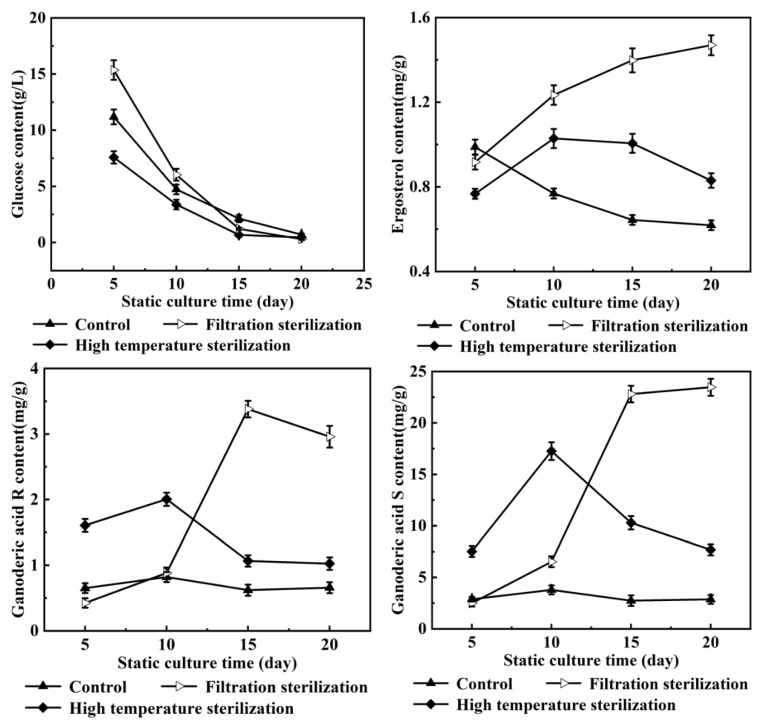

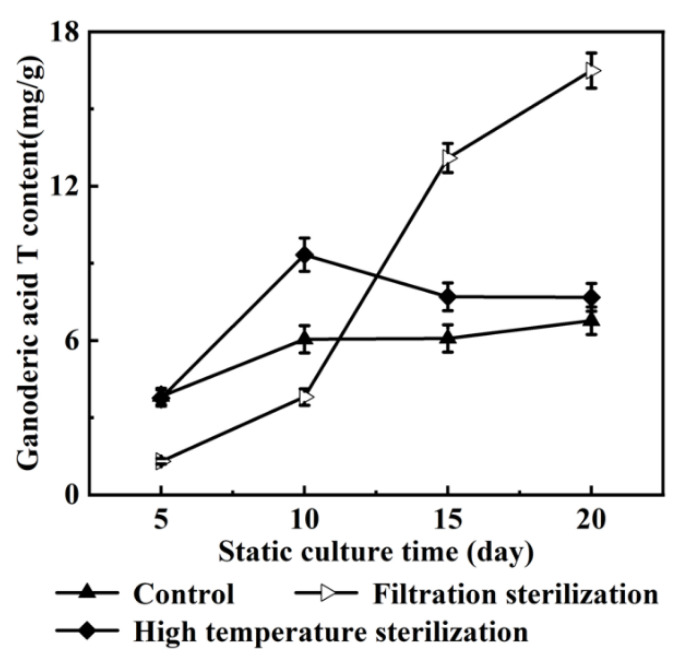
Substrate and product contents of *G. lucidum* submerged fermentation-static culture under different oleic acid addition methods.

The consumption of glucose, the synthesis of the by-product ergosterol and the target product (ganoderic acids R, S and T) in the two-stage fermentation culture of *G. lucidum* were analyzed during the fermentation process. As shown in Figure 1, the consumption of glucose under the conditions of adding high-temperature-sterilized oleic acid and filter-sterilized oleic acid were consistent with that of the control group, and the glucose was basically consumed on the 20th day of static culture. However, the synthesis of ergosterol was quite different. Under the condition of adding high-temperature-sterilized oleic acid, the synthesis of ergosterol increased in advance and then decreased, with a maximum value of 1.03 mg/g on the 10th day of static culture. The content of ergosterol was positively correlated with the culture time under the condition of adding filter-sterilized oleic acid, and reached the maximum value of 1.47 mg/g on the 20th day of culture. The ergosterol content in the control group was the highest on the 5th day of static culture, which was 0.99 mg/g. Compared with the control group, the content of ganoderic acids R, S and T was quite different. Under the condition of adding high-temperature-sterilized oleic acid, the ganoderic acids R, S and T reached the maximum of 2.00 mg/g, 17.26 mg/g and 9.33 mg/g on the 10th day of static culture, which were 1.44 times, 3.55 times and 0.38 times higher than the control of 0.82 mg/g (the 10th day of static culture), 3.79 mg/g (the 10th day of static culture) and 6.77 mg/g (the 20th day of static culture), respectively. The highest results of the ganoderic acids R, S and T in the filter-sterilized oleic acid group were 3.38 mg/g (15 days), 23.46 mg/g (20 days) and 16.49 mg/g (20 days), respectively, which were 3.11 times, 5.19 times and 1.44 times higher, respectively, than those of the control group.

It can be seen from Figure 1, that both methods of oleic acid addition are beneficial in increasing ganoderic acids R, S and T’s content. In order to further interpret the effects of oleic acid on these ganoderic acids’ biosynthesis under two different addition methods, the specific regulation methods of oleic acid on the liquid fermentation metabolism of *G. lucidum* mycelium were analyzed through a metabolic flux analysis.

### 3.2. Metabolic Flux Balance Model of Ganoderic Acids R, S and T

**Figure 2:**
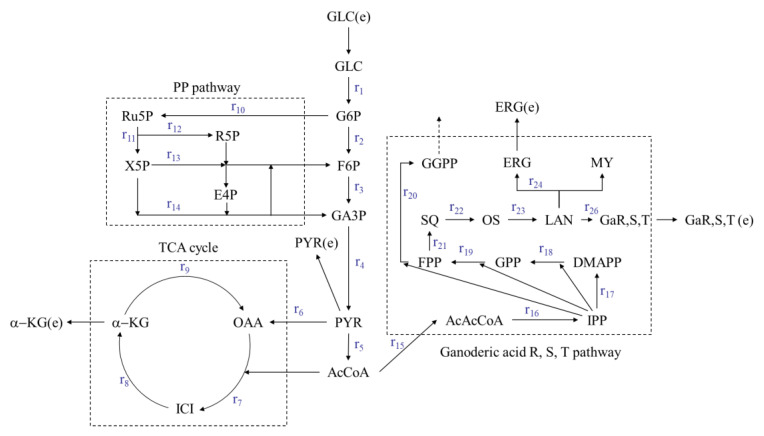
The metabolic network of ganoderic acids R, S and T synthesis by *G. lucidum,* submerged fermentation-static culture. AcAcCoA—acetoacetyl coenzyme A; AcCoA—Acetyl Coenzyme A; DMAPP—Dimethylallyl pyrophosphate; E4P—Erythrose-4-phosphate; ERG—Ergosterol; ERG (e)—Extracellular ergosterol; F6P—fructose-7-phosphate; FADH2—Reduced Flavin Dinucleotide; FPP—Farnesylpyrophosphate; G6P—Glucose-6-phosphate; GA3P—glyceraldehyde 3-phosphate; Ga R, S, T—Ganoderic acid R, Ganoderic acid S, Ganoderic acid T; GGPP—geranylgeranyl pyrophosphate; GLC—Glucose; GLC (e)—Extracellular glucose; GPP—Geranyl pyrophosphate; ICI—Isocitric acid; IPP—Isopentenyl pyrophosphate; LAN—Lanosterol; MY—Mycosterol; NADH—Nicotinamide adenine dinucleotide; OAA—Oxaloacetic acid; OS—2,3-Oxidosqualene; PYR—Pyruvate; PYR (e)—Extracellular pyruvate; R5P—Ribose-5-phosphate; Ru5P—ribulose 5-phosphate; SQ—Squalene; X5P—Xylulose phosphate; α-KG—α-ketoglutaric acid; α-KG (e)—Extracellular α-ketoglutaric acid.

Based on the relevant literature [19,20,21,22], a metabolic network of the synthesis of the ganoderic acids R, S and T in a submerged fermentation-static culture was established (Figure 2). The metabolic network consisted of a glycolysis pathway (EMP), a tricarboxylic acid cycle (TCA), a pentose phosphate pathway (PP) and a ganoderic acids (R, S and T) synthesis pathway (GAP). Detailed metabolic reaction equations and metabolic node reaction rate equations are shown in Table 1. The metabolic network included 26 unknown reaction rates and 24 reaction equations with a degree of freedom of two; therefore linear equations can be solved merely by measuring just two-step reaction rates. In this study, the main variables measured were as follows: the glucose consumption rate and the ergosterol production rate.

### 3.3. Effects of Oleic Acid Addition Methods on Metabolic Flux Distribution of Ganoderic Acids R, S and T’s Biosynthesis

**Figure 3:**
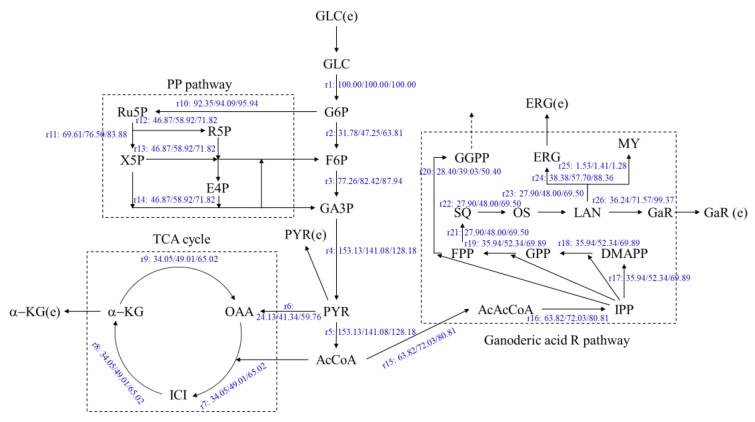
Metabolic flux distribution of ganoderic acid R, synthesized by submerged fermentation-static culture under two methods of oleic acid addition. Left: Control (the 10th day of static culture); Middle: Filter-sterilized oleic acid (15th day of static culture); Right: High-temperature-sterilized oleic acid (10th day of static culture).

**Figure 4:**
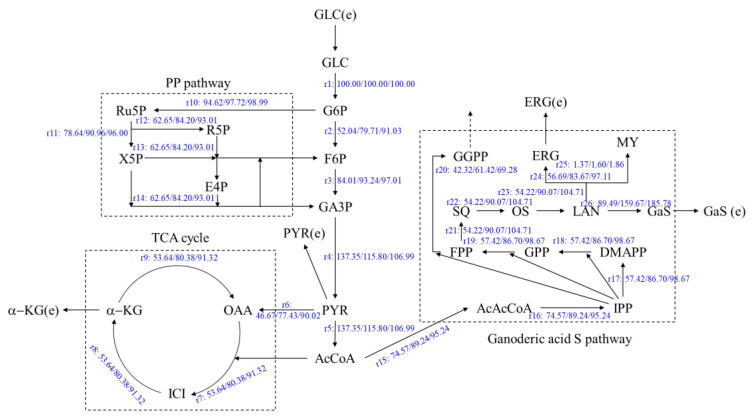
Metabolic flow distribution of ganoderic acid S, synthesized by submerged fermentation-static culture under two methods of oleic acid addition. Left: Control (the 10th day of static culture); Middle: Filtration-sterilization oleic acid (20 days of static culture); Right: High-temperature-sterilization oleic acid (10 days of static culture).

**Figure 5:**
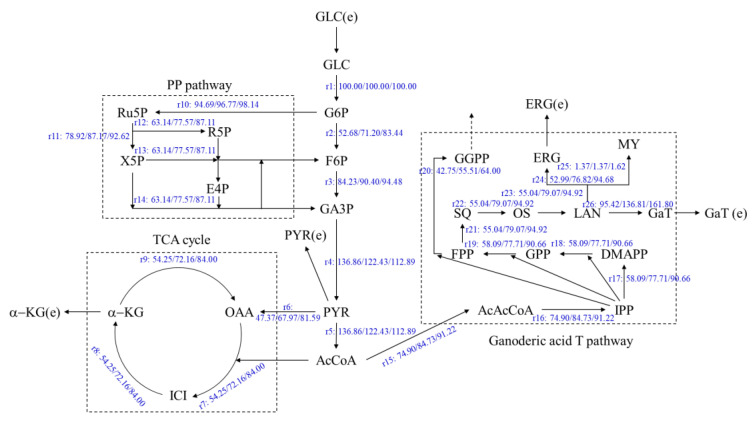
Metabolic flow distribution of ganoderic acid T, synthesized by submerged fermentation-static culture under two methods of oleic acid addition. Left: Control (the 20th day of static culture); Middle: Filtration-sterilization oleic acid (20 days of static culture); Right: High-temperature-sterilization oleic acid (10 days of static culture).

The metabolic flux of the ganoderic acids R, S and T–synthesized from *G. lucidum* with two oleic acid addition methods and no addition group in two stages of submerged fermentation-static culture, under the highest target products yields–were analyzed. The concentration of glucose and ergosterol in the fermentation were determined. The metabolic flow distribution of the ganoderic acids R, S and T were obtained by linear programming with MATLAB software. The results are shown in Figure 3, Figure 4 and Figure 5.

As shown in Figure 3, the flux at the acetoacetyl-CoA (a key metabolic node to enter into the ganoderic acid synthesis) to the GARP (r15) of under filter-sterilized and high-temperature-sterilized oleic acid addition, were 72.03 and 80.81, which increased by 12.86% and 26.62%, respectively, compared with the control fermentation process of 63.82. The flux to the lanosterol nodes(r23) were 48.00 and 69.50, which increased by 72.05% and 149.15%, respectively, compared with the control of 27.90. The flux to the ergosterol node(r24) were 57.70 and 88.36, which were enhanced by 50.33% and 130.22%, respectively, compared with the control of 38.38. The increasement of the ergosterol flux promoted the synthesis of ergosterol, which was consistent with the ergosterol contents in Figure 2 (due to the enhancement of this node, the synthesis of ergosterol was promoted). The flux (r26) to the ganoderic acid R node were 71.57 and 99.37, which increased by 97.48% and 174.19%, respectively, compared with the control (36.24). Therefore, the addition of oleic acid can improve the metabolic flux through the above key nodes and can promote the synthesis of ganoderic acid R.

As shown in Figure 4, the flux at the acetoacetyl-CoA to the GASP (r15) of under filter-sterilized and high-temperature-sterilized oleic acid addition, were 89.24 and 95.24, increased by 19.67% and 27.72%, respectively, compared with the control fermentation process of 74.57. The flux to the lanosterol nodes (r23) were 90.07 and 104.71, which increased by 66.13% and 93.14%, respectively, compared with the control (54.21). The flux to the ergosterol node(r24) were 83.67 and 97.11, which were enhanced by 47.60% and 71.32%, respectively, compared with the control of 56.69. The increasement of the ergosterol flux promoted the synthesis of ergosterol, which also corresponded to the ergosterol contents in Figure 2. The flux (r26) to the ganoderic acid S node were 159.67 and 185.78, which increased by 78.42% and 107.59%, respectively, compared with the control (89.49). Therefore, the addition of oleic acid can improve the metabolic flux through the above key nodes and can promote the synthesis of ganoderic acid S.

As shown in Figure 5, the flux at the acetoacetyl-CoA to the GATP (r15) of under filter-sterilized and high-temperature-sterilized oleic acid addition, were 84.72 and 91.22, increased by 13.11% and 21.78%, respectively, compared with the control fermentation process of 74.90. The flux to the lanosterol node(r23) were 70.07 and 94.91, increased by 43.66% and 72.46%, respectively, compared with the control of 55.04. The flux to ergosterol node(r24) were 76.82 and 94.68, which were enhanced by 44.98% and 78.67%, respectively, compared with the control of 52.99. The increasement of the ergosterol flux promoted the synthesis of ergosterol, which also corresponded to the ergosterol contents in Figure 2. The flux (r26) to the ganoderic acid T node were 136.81 and 161.80, which increased by 43.38% and 69.56%, respectively, compared with the control (95.42). Therefore, the addition of oleic acid can improve the metabolic flux through the above key nodes and can promote the synthesis of ganoderic acid T.

### 3.4. Effects of Oleic Acid Addition Methods on Metabolic Pathway Flux in Ganoderic Acids R, S and T’s Biosynthesis

To further compare the effects of two oleic acid addition methods on the flux direction of the metabolic pathways in a submerged fermentation-static culture of *G. lucidum*, the distribution of the metabolic flux was calculated according to their metabolic pathways, respectively. The results are shown in Table 2. From these results, it can be seen that oleic acid added by filtration sterilization and high temperature sterilization promoted all of the four metabolic pathways in different degrees. Among them, the influence on the ganoderic acid R synthesis pathway was the greatest, which increased by 45.10% and 93.35%, respectively; it had the least effect on the glycolysis pathway, which increased by 2.37% and 4.91%, respectively. The improvement rates of the tricarboxylic acid cycle were 17.91% and 37.07%, respectively. The improvement rates of the pentose phosphate pathway were 14.80% and 30.64%, respectively. These results implied that the addition of oleic acid enhances the distribution of carbon metabolic flow as a whole, improves the flow direction in each pathway and contributes to enhancing the accumulation of ganoderic acid R. Further analysis of the metabolic flow distribution of ganoderic acid S and ganoderic acid T showed that the oleic acid added by two sterilization methods enhanced the metabolic flow of the four metabolic pathways in varying degrees. The enhancement of the metabolic flow in the biosynthesis of ganoderic acid R was increased by 80.18% and 165.97%, respectively; the enhancement of the metabolic flow in the biosynthesis of ganoderic acid S was increased by 102.84% and 144.89%, respectively; and the enhancement of the metabolic flow in the biosynthesis of ganoderic acid T was increased by 68.30% and 113.44%, respectively.

## 4. Discussion

In the liquid submerged fermentation of *G. lucidum*, the downstream metabolic pathway of triterpenes synthesis is still unclear, it is difficult to improve the yield of triterpenes by means of genetic engineering or metabolic pathway engineering, and the low production of triterpenes has always been the bottleneck problem restricting the development of the industry. In view of this situation, we have cast a new light on the metabolic flux analysis method. Combined with previous studies, we found that adding oleic acid in fermentation can greatly improve the ganoderic acids R, S and T’s contents. In order to further explore the effects of oleic acid, added by two sterilization methods, on the biosynthesis of ganoderic acids R, S and T, this study explained the flow direction of carbon metabolic and the changes of by-products in the synthesis of ganoderic acids R, S and T by means of metabolic flow distribution.

Metabolic flux analysis is a method of stoichiometry analysis with measured data. Based on the mass balance law and pseudo-steady-state assumption, it calculates the intracellular, microscopic and unmeasurable metabolic flux by using acquirable information, such as observable state variables and chemical reaction formulas in the metabolic network [23,24]. Metabolic flow distribution analysis has many applications in the metabolic synthesis of goal products by bacteria and fungi. Niu [25] constructed a metabolic network of *Escherichia coli* fermentation to promote glycerol to produce L-methionine and found that L-methionine was mainly obtained from the pathway of phosphoenolpyruvate (PEP) to oxaloacetic acid (OAA). Enhancing the HMP pathway can provide a large amount of NADPH, while increasing the dissolved oxygen level can promote the synthesis of goal products. Tomàs-Gamisans et al. [26] found that glycerol as a sole carbon source could promote amino acid production by *Pichia pastoris,* by metabolic flux analysis. Hayakawa et al. [27] conducted a metabolic model of adding ethanol to promote S-adenosyl-L-methionine production in *Saccharomyces cerevisiae* and found that the metabolic flux levels of the tricarboxylic acid cycle and glyoxylate shunt in the ethanol culture were significantly higher than that in the glucose culture, and more ATP was produced from ethanol by oxidative phosphorylation. However, to our knowledge, there are no reports in the existing literature on this type of research, due to the complexity of the metabolites of edible and medicinal fungi and the lack of in-depth research on metabolic networks.

In this study, we constructed a metabolic network of carbon metabolic flow, which consisted of a glycolysis pathway (EMP), a tricarboxylic acid cycle (TCA), a pentose phosphate pathway (PP) and ganoderic acids R, S and T synthesis pathways (GAP). Glucose was used as a substrate in the metabolic pathway, and the effects of oleic acid on each pathway were investigated before and after the oleic acid addition. The results showed that additional oleic acid promoted the pentose phosphate pathway and the tricarboxylic acid cycle and strengthened the energy supply. At the same time, it was found that oleic acid mainly enhanced the synthesis of lanosterol, weakened the synthesis of the by-product mycosterol, and then promoted the synthesis of ganoderic acid. It was found that although the metabolic pathway of lanosterol was strengthened, the metabolic flow of the by-product ergosterol was also strengthened under the two methods of oleic acid addition. The metabolic flow (r24) of ergosterol with the addition of high-temperature-sterilized oleic acid was much higher than that with the addition of filter-sterilized oleic acid. This is because 1.35% of filter-sterilized oleic acid was added at the 7th hour of fermentation, while 1.21% high-temperature-sterilized oleic acid was added at the 32nd hour of fermentation. Although the addition time and the amount of oleic acid were different, they promoted the differences in the flow direction of the mycelium metabolism together, which led to the differences in the ganoderic acids’ content. According to the correlation analysis from the results in Table 2 and the results of the contents of the three ganoderic acids in Figure 1, under the condition of adding filter-sterilized oleic acid, the increasing rate of the total metabolic flow was less than that of adding high-temperature-sterilized oleic acid. However, the content of ganoderic acids R, S and T in the former was higher than that in the latter, and the content of the by-product ergosterol was much higher than that in the latter. The reason for this might be due to the influence of the other by-products (except ergosterol) on the metabolism of the ganoderic acids R, S and T. In the follow-up study, if the metabolic flow of ergosterol and the other by-products’ synthesis are further blocked by strain transformation or process optimization, then the metabolic flow of its by-product synthesis would be inhibited. It will be beneficial to strengthen the metabolic flow of ganoderic acid synthesis and then increase the yield of ganoderic acid.

## Figures and Tables

**Table 1:** Metabolic reaction equations and metabolic node reaction rate equations of ganoderic acids R, S and T’s synthesis from submerged fermentation-static culture of *G. lucidum*.

Metabolic Reaction Equations	Metabolic Node Reaction Rate Equations
Glycolysis pathway (EMP):	(1) GLC: GLC(e) − r1 = 0
r1: GLC + ATP → G6P + ADP	(2) G6P: r1 − r2 − r10 = 0
r2: G6P → F6P	(3) F6P: r2 − r3 + r13 + r14 = 0
r3: F6P + ATP → 2GA3P + ADP	(4) GA3P: 2r3 − r4 + r14 = 0
r4: GA3P + NAD + PI + 2ADP → PYR + NADH + 2ATP	(5) PYR: r4 − r5 − r6 = 0
Tricarboxylic acid cycle (TCA):	(6) AcCoA: r5 − r7 − 2r15 − r16 = 0
r5: PYR + CoA + NAD → AcCoA + NADH + CO_2_	(7) ICI: r7 − r8 = 0
r6: PYR + ATP + CO_2_ → OAA + ADP + PI	(8) α-KG: r8 − r9 = 0
r7: OAA + AcCoA → ICI + CoA	(9) OAA: r6 − r7 + r9 = 0
r8: ICI + NADP → α − KG + NADPH + CO_2_	(10) Ru5P: r10 − r11 − r12 = 0
r9: α − KG + 2NAD + FAD + ADP + PI → OAA + 2NADH + FADH_2_ + ATP + CO_2_	(11) X5P: r11 − r13 − r14 = 0
Pentose phosphate pathway (PP):	(12) R5P: r12 − r13 = 0
r10: G6P + 2NADP → Ru5P + 2NADPH + CO_2_	(13) E4P: r13 − r14 = 0
r11: Ru5P → X5P	(14) CoA: -r5 + r7 + r15 + 2r16 = 0
r12: Ru5P → R5P	(15) AcAcCoA: r15 − r16 = 0
r13: X5P + R5P → F6P + E4P	(16) IPP: r16 − r17 − r18 − r19 − r20 = 0
r14: X5P + E4P → F6P + GA3P	(17) DMAPP: r17 − r18 = 0
Ganoderic acids R, S, T synthesis pathway (GAP):	(18) GPP: r18 − r19 = 0
r15: 2AcCoA → AcAcCoA + CoA	(19) FPP: r19 − r20 − 2r21 = 0
r16: AcAcCoA + AcCoA + 2NADPH + 2H^+^ + 3ATP + H_2_O → IPP + 2CoA + 2NADP^+^ + 3ADP + PI + CO_2_	(20) SQ: r21 − r22 = 0
r17: IPP → DMAPP	(21) OS: r22 − r23 = 0
r18: DMAPP + IPP → GPP + PPI	(22) LAN: r23 − r24 − r25 − r26 = 0
r19: GPP + IPP → FPP + PPI	(23) ERG: r24 − ERG(e) = 0
r20: FPP + IPP → GGPP + PPI	(24) GaR: r26 − GaR(e) = 0
r21: 2FPP + NADPH + H^+^ → SQ + NADP^+^ + 2PPI	GaS: r26 − GaS(e) = 0
r22: SQ + O_2_ + NADPH + H^+^ → OS + NADP^+^ + H_2_O	GaT: r26 − GaT(e) = 0
r23: OS → LAN	
r24: LAN → ERG	
r25: LAN → MY	
r26: LAN → GaR/GaS/GaT	

**Table 2:** Statistics of metabolic pathway flow direction of ganoderic acids R, S and T, synthesized by submerged fermentation-static culture under two methods of oleic acid addition.

Metabolic Pathway	Control	Filter-Sterilized Oleic Acid		High-Temperature-Sterilized Oleic Acid	
		Flow	Increasing Rate (%)	Flow	Increasing Rate (%)
Ganoderic acid R					
EMP	362.17	370.75	2.37	379.93	4.91
TCA	279.42	329.46	17.91	383.00	37.07
PP	302.57	347.36	14.80	395.28	30.64
Ganoderic acid R synthesis pathway (GaRP)	423.70	614.77	45.10	819.22	93.35
Ganoderic acid S					
EMP	373.41	388.75	4.11	395.02	5.79
TCA	344.94	434.39	25.93	470.98	36.54
PP	361.22	441.27	22.16	474.03	31.23
Ganoderic acid S synthesis pathway (GaSP)	673.91	1015.16	50.64	1154.66	71.34
Ganoderic acid T					
EMP	373.76	384.03	2.75	390.82	4.57
TCA	346.98	406.87	17.26	446.47	28.67
PP	363.05	416.64	14.76	452.09	24.53
Ganoderic acid T synthesis pathway (GaTP)	681.70	910.28	33.53	1061.26	55.68

## Data Availability

Not applicable.
